# Cost-effectiveness comparison between palpation- and ultrasound-guided thyroid fine-needle aspiration biopsies

**DOI:** 10.1186/1472-6823-9-14

**Published:** 2009-05-16

**Authors:** Ahmet Selçuk Can

**Affiliations:** 1 Division of Endocrinology and Metabolism, Department of Medicine, Private Gayrettepe Florence Nightingale Hospital, Gayrettepe, Beşiktaş, Istanbul, Turkey

## Abstract

**Background:**

The aim of this study is to perform a cost-effectiveness comparison between palpation-guided thyroid fine-needle aspiration biopsies (P-FNA) and ultrasound-guided thyroid FNA biopsies (USG-FNA).

**Methods:**

Each nodule was considered as a case. Diagnostic steps were history and physical examination, TSH measurement, Tc^99m ^thyroid scintigraphy for nodules with a low TSH level, initial P-FNA versus initial USG-FNA, repeat USG-FNA for nodules with initial inadequate P-FNA or USG-FNA, hemithyroidectomy for inadequate repeat USG-FNA. American Thyroid Association thyroid nodule management guidelines were simulated in estimating the cost of P-FNA strategy. American Association of Clinical Endocrinologists guidelines were simulated for USG-FNA strategy. Total costs were estimated by adding the cost of each diagnostic step to reach a diagnosis for 100 nodules. Strategy cost was found by dividing the total cost to 100. Incremental cost-effectiveness ratio (ICER) was calculated by dividing the difference between strategy cost of USG-FNA and P-FNA to the difference between accuracy of USG-FNA and P-FNA. A positive ICER indicates more and a negative ICER indicates less expense to achieve one more additional accurate diagnosis of thyroid cancer for USG-FNA.

**Results:**

Seventy-eight P-FNAs and 190 USG-FNAs were performed between April 2003 and May 2008. There were no differences in age, gender, thyroid function, frequency of multinodular goiter, nodule location and diameter (median nodule diameter: 18.4 mm in P-FNA and 17.0 mm in USG-FNA) between groups. Cytology results in P-FNA versus USG-FNA groups were as follows: benign 49% versus 62% (p = 0.04), inadequate 42% versus 29% (p = 0.03), malignant 3% (p = 1.00) and indeterminate 6% (p = 0.78) for both. Eleven nodules from P-FNA and 18 from USG-FNA group underwent surgery. The accuracy of P-FNA was 0.64 and USG-FNA 0.72. Unit cost of P-FNA was 148 Euros and USG-FNA 226 Euros. The cost of P-FNA strategy was 534 Euros and USG-FNA strategy 523 Euros. Strategy cost includes the expense of repeat USG-FNA for initial inadequate FNAs and surgery for repeat inadequate USG-FNAs. ICER was -138 Euros.

**Conclusion:**

Universal application of USG-FNA for all thyroid nodules is cost-effective and saves 138 Euros per additional accurate diagnosis of benign versus malignant thyroid nodular disease.

**Trial registration:**

ClinicalTrials.gov, NCT00571090

## Background

There are two thyroid nodule management guidelines with global recognition: first is the management guidelines for patients with thyroid nodules and differentiated thyroid cancer by American Thyroid Association (ATA) Guidelines Taskforce and the second one is the American Association of Clinical Endocrinologists (AACE) and Associazione Medici Endocrinologi medical guidelines for clinical practice for the diagnosis and management of thyroid nodules. After identification of a thyroid nodule that is more than 10–15 mm, ATA recommends measuring serum TSH level. If TSH is low, a thyroid scan is next. For a hot thyroid nodule, evaluation and treatment for hyperthyroidism is recommended. If the nodule is not hot, the algorithm points to diagnostic ultrasonography. The flowchart directs again to diagnostic ultrasonography for euthyroid and hypothyroid patients with thyroid nodules. If a nodule is ultrasonographically confirmed, a cytological evaluation is next. ATA recommends ultrasound-guided thyroid fine-needle aspiration biopsy (USG-FNA) for nodules with more than 50% cystic component and for nodules located in posterior parts of the thyroid gland. Otherwise either palpation-guided (P-FNA) or USG-FNA is next [1]. In contrast, as indicated in the abstract of their guideline, AACE recommends universal application of USG-FNA for all thyroid nodules ≥ 10 mm, except for subjects with a low serum TSH level and a hot nodule, in whom FNA is not indicated [2]. Except for minor changes in the sequence of diagnostic steps, ATA and AACE thyroid nodule management guidelines differ only in the utilization of ultrasound guidance. All previous studies show that USG-FNA has higher sensitivity, specificity and accuracy than P-FNA [3-11]. A barrier for universal recommendation of USG-FNA might be its higher initial cost [12]. The aim of this report is to compare cost-effectiveness between palpation- and ultrasound-guided thyroid fine-needle aspiration biopsies from a perspective of ATA versus AACE thyroid nodule management guidelines.

## Methods

This is a secondary data analysis of a study registered to ClinicalTrials.gov with Identifier NCT00571090. The aim of the study was to identify ultrasonographic features that predict malignant thyroid nodules. The primary data from this study has not been published. Data relevant to cost-effectiveness comparison are reported here. FNAs were performed as an outpatient procedure in Gayrettepe and Şişli Florence Nightingale Hospitals, two private hospitals affiliated with Istanbul Science University and authors' private medical office in Şişli, all located in Istanbul. Turkish Cardiology Foundation owns and operates Istanbul Science University and Group Florence Nightingale Hospitals in Istanbul. These health care facilities are secondary care centers for thyroid disorders, not tertiary care referral centers.

The clinical information and cytology results of all consecutive patients who underwent FNA biopsy of thyroid nodules in above-mentioned outpatient endocrinology clinics were instantaneously recorded in a computerized database on the day of the FNA biopsy. This study was not a retrospective chart review. All patients were examined by ASC, an endocrinologist who also performed all thyroid FNA biopsies between April 2003 and May 2008. Thyroid function tests and thyroid ultrasonography were routinely obtained. Subjects were classified into euthyroid, hypothyroid (TSH>4.20 μIU/ml) and hyperthyroid (TSH<0.27 μIU/ml) categories according to the results of thyroid function tests. The location, three dimensional diameters, echo structure (solid, mixed or cystic), echogenecity (hypoechoic, isoechoic or hyperechoic) and other ultrasonographic features of thyroid nodules were recorded. A nodule was classified as cystic, if its area was 90–100% cystic. Thyroid scintigraphy with Technetium^99m ^was obtained for subjects with a low TSH level. All subjects consented to FNA biopsy in accordance with the hospital bylaws. Institutional Review Board approval from Istanbul Science University Ethics Committee was asked and granted in July 2007 (Number 2007/006). As thyroid FNAs were performed in routine clinical care without any investigational procedure, the Ethics Committee did not ask for signed informed consent for subjects enrolled prior to approval of the study. Subjects admitted after July 2007 signed informed consent for the study.

Each thyroid nodule was counted as a case in this report. Three-hundred and thirty-nine FNAs were performed between April 2003 and May 2008. Nineteen FNAs for nonpalpable nodules and 16 FNAs for nodules <10 mm were excluded. There were 19 nodules with a low TSH level but without a thyroid scintigraphy and these were also excluded. ATA designates cystic nodules as an indication for USG-FNA [1]. AACE recommends USG-FNA for all nodules, including cystic ones [2]. As there was no disagreement on the utility of ultrasound guidance for cystic nodules between guidelines, 17 cystic nodules were excluded from the analysis. There were 268 thyroid nodules for comparison. One-hundred and ninety USG-FNAs were performed to palpable thyroid nodules with a SonoSite 180 plus hand-carried ultrasound system and a L38/10-5 MHz transducer between March 2004 and May 2008, starting with the acquisition of the ultrasound system. Both the ultrasound system and the transducer were manufactured by SonoSite Incorporation, located in Bothell, WA, USA. A published standard technique was used for USG-FNA [13]. Seventy-eight P-FNAs were performed to palpable thyroid nodules with a previously published technique [14] between April 2003 and February 2008, at times when the ultrasound system was not available for FNA biopsy. Allocation to P-FNA and USG-FNA groups was not random. The FNAs were done by palpation-guidance until the ultrasonography system was purchased. Then USG-FNA was routinely performed. P-FNA was still performed after the acquisition of ultrasonography system, at times when the ultrasonography machine was temporarily unavailable in the above-mentioned outpatient endocrinology clinics. On-site microscopic adequacy was not determined. Local anesthesia with 2% lidocaine was routinely administered for both P- and USG-FNAs. All FNA biopsies were performed with either 22 or 26 gauge needles. Twenty-two gauge needles are long; 26 gauge needles are short. If the nodule is located anteriorly or in the isthmus a 26 G needle, if posteriorly a 22 G needle was used. Two needle aspirations were carried out for each nodule. If no material was seen on the slides to plain eye without using an optical instrument, up to four aspirations were performed. Half of the smears were air-dried and stained with May-Grünwald-Giemsa and the other half were alcohol-fixed and stained with Hematoxylene-Eosine or Papanicolaou. Cytological diagnoses were categorized as malignant, benign, indeterminate and inadequate [1,14]. Six clusters of benign cells in at least two slides constituted adequate material for cytological diagnosis. Each cluster was composed of at least 15 cells. The smears that do not meet these criteria were assigned into inadequate category. Indeterminate samples included a pattern of follicular or Hurthle cell neoplasm or aspects of atypia suggestive, but not conclusive of the presence of a malignant neoplasm [14].

### Statistical methods

Continuous variables were presented as means ± standard deviations and Student's *t *test was used for comparison. As maximal nodule diameter had a positively skewed distribution, its logarithmic transformation was used in Student's *t *test. Categorical variables were presented as percentages and χ^2 ^test was used for comparison. As the expected frequencies of malignant results were less than five in both groups, each cytology category in P-FNA and USG-FNA groups was compared after collapsing the rest of the categories. Fisher's exact test was employed when the expected frequencies were less than five. Benign and inadequate cytology results were categorized into negative tests, as surgery is not recommended for these conditions. Malignant and indeterminate FNA cytology results were categorized into positive tests, as surgery is recommended to patients with such results. Nodules with benign or inadequate cytology and benign surgical histopathology result were classified as true negatives. Nodules with benign or inadequate cytology and malignant surgical histopathology result were classified as false negatives. Nodules with malignant or indeterminate cytology and malignant surgical histopathology result were classified as true positives. Nodules with malignant or indeterminate cytology and benign surgical histopathology result were classified as false positives. The accuracy of the test was calculated by dividing the sum of true positives and true negatives to the sum of true positives, false positives, false negatives and true negatives.

### Cost of thyroid fine-needle aspiration strategies

Health care system of Republic of Turkey is dichotomized to state (government) and private sector. According to Turkish Ministry of Health, 10% of the population receives services from private health care sector in Turkey [15]. This study was performed in a private health care setting. The minimum fee for medical and laboratory procedures in private health care system is determined by the City Chamber of Physicians, a non-governmental organization elected by physicians. Istanbul Chamber of Physicians' minimum fees are used in this study and are shown in table 1. Private health care sector charges the minimum fee or usually up to two-fold of it. The minimum fee in table 1 is valid for all private health care facilities in Istanbul. These were actual billed and collected fees obtained from Gayrettepe Florence Nightingale Hospital Patients' Accounts Office. Derived from patients' bills, subtotal hemithyroidectomy fee includes all costs including one night hospital stay but excludes the cost of harmonic scalpel, an expensive device that was used by some surgeons in our hospital. ATA and AACE thyroid nodule management guidelines were constructed using actual frequencies from this study and actual costs from Table 1. The cost of each diagnostic step was summed until a final cytological or pathological diagnosis was made for one hundred nodules. Malignant, benign and indeterminate cytological results were final diagnoses. For inadequate smears, USG-FNA was the next step whether or not initial FNA was palpation- or ultrasound-guided. If two consecutive thyroid FNAs were inadequate, a hemithyroidectomy was the final procedure to reach a diagnosis [16]. Cost per nodule was calculated by dividing the sum of costs for one hundred nodules to 100. Incremental cost-effectiveness ratio (ICER) was estimated to find the amount of more spending versus more saving to achieve one additional correct diagnosis of benign versus malignant thyroid nodule, if one modality is used over the other. ICER [17] was calculated with the following formula:

**Table 1:** Cost of diagnostic steps recommended by thyroid nodule management guidelines

Diagnostic Step	Cost
History and physical exam	€39.03
Measurement of TSH	€23.42
Thyroid ultrasonography	€39.03
Tc^99m ^thyroid scintigraphy	€93.66
Palpation-guided FNA	€148.30
Ultrasound-guided FNA	€226.35
Subtotal hemithyroidectomy	€1545.77

FNA: thyroid fine-needle aspiration biopsy. The exchange rate of European Central Bank on July 4^th ^2008 was used to convert costs in New Turkish Liras to Euros. 8% value addition tax is included. Costs are from Turkish private health care sector in Istanbul.



*c*USG-FNA is the cost of ultrasound-guided thyroid FNA strategy according to AACE thyroid nodule management guideline to achieve a diagnosis for one nodule, *c*P-FNA is the cost of palpation-guided thyroid FNA strategy according to ATA thyroid nodule management guideline to achieve a diagnosis for one nodule, *a*USG-FNA is the accuracy of ultrasound-guided thyroid FNA and *a*P-FNA is the accuracy of palpation-guided FNA.

## Results

In the palpation group, 36 patients had a solitary nodule and 21 patients had two nodules, hence 78 FNAs were performed by palpation. In the ultrasound-guided group, 102 patients had a solitary nodule, 38 patients had two nodules and 4 patients had three nodules, hence 190 FNAs were performed with ultrasound-guidance. The frequency of multinodular goiter was 37% in P-FNA and 29% in USG-FNA group (p = 0.29). The presence of multinodular goiter was determined by ultrasonography on all subjects. There were no differences in baseline characteristics between P-FNA and USG-FNA groups (table 2). The median maximal nodule diameter was 18.4 mm, the 25^th ^percentile 12.5 mm and 75^th ^percentile 25.8 mm in the P-FNA group. The median maximal nodule diameter was 17.0 mm, the 25^th ^percentile 14.0 mm and 75^th ^percentile 22.8 mm in the USG-FNA group. There were no differences in nodule diameter between groups (table 2). In the USG-FNA group, significantly more subjects had benign cytology results and significantly less number of subjects had inadequate smears compared to P-FNA group. In the P-FNA group, the echo structure of nodules with an inadequate result (*n *= 33) was 39.4% (*n *= 13) solid and 60.6% (*n *= 20) mixed. The echogenecity of nodules with an inadequate result was 42.4% (*n *= 14) hypoechoic, 36.4% (*n *= 12) isoechoic, 12.1% (*n *= 4) hyperechoic, 9.1% (*n *= 3) both hypoechoic and isoechoic in the P-FNA group. In the USG-FNA group, the echo structure of nodules with an inadequate result (*n *= 55) was 65.5% (*n *= 36) solid and 34.5% (*n *= 19) mixed. The echogenecity of nodules with an inadequate result was 47.3% (*n *= 26) hypoechoic, 41.8% (*n *= 23) isoechoic, 5.5% (*n *= 3) hyperechoic, 3.6% (*n *= 2) both hypoechoic and isoechoic, 1.8% (*n *= 1) both hypoechoic and hyperechoic in the USG-FNA group. Significantly more nodules with an inadequate result had a mixed echo structure in the P-FNA group (χ^2 ^test, p = 0.02), but there were no differences in echogenecity between P-FNA and USG-FNA groups (χ^2 ^or Fisher's exact test, p > 0.05). The frequency of malignant or indeterminate diagnoses did not differ between groups. Eleven nodules from P-FNA and 18 from USG-FNA group underwent thyroid surgery (tables 3 and 4). In the palpation-guided group, surgical histopathology showed two papillary thyroid carcinomas. One of the papillary carcinomas was found to be malignant in prior cytology and was a true positive. The other papillary carcinoma was reported as benign in P-FNA cytology and was a false negative. There were nine nodules that were benign in surgical histopathology in the palpation group. In prior cytology, four of them were benign and two of them were inadequate, so there were six true negatives. Two of the histopathologically benign nodules were categorized as indeterminate and one of them as malignant in prior cytology, so there were three false positives. The nodule with the malignant cytology and benign pathology was a benign adenomatous nodule with lymphocytic thyroiditis, as well as the first nodule with the indeterminate result. The other indeterminate lesion was a micro- and macrofollicular colloid nodule. Both indeterminate nodules had Hurthle cell metaplasia in histopathology. Cross-tabulation of cytology and pathology results in the P-FNA group was summarized in table 3. The sensitivity of P-FNA was 0.50, specificity 0.66 and accuracy 0.64. In the ultrasound-guided group, surgical histopathology showed five papillary, one follicular and four medullary thyroid carcinomas. Eight of these were found to be malignant in prior cytology and were true positives. One medullary carcinoma was reported as benign and the other as inadequate in prior USG-FNA cytology, so there were two false negatives. There was a high prevalence of medullary thyroid cancer in the USG-FNA group. The medullary thyroid cancer patients were not relatives and there was no single cluster of familial medullary thyroid carcinoma in the USG-FNA group. There were eight nodules that were benign in surgical histopathology in USG-FNA group. In prior cytology, two of them were benign and three were inadequate, so there were five true negatives. Three histopathologically benign nodules were categorized as indeterminate in prior cytology and were false positives. The cytologically indeterminate lesions were adenomatous nodules with lymphocytic thyroiditis in surgical histopathology. Cross-tabulation of cytology and pathology results in the USG-FNA group was summarized in table 4. The sensitivity of USG-FNA was 0.80, specificity 0.63 and accuracy 0.72.

**Table 2:** Comparison of baseline characteristics and cytology results between palpation-guided and ultrasound-guided thyroid fine-needle aspiration biopsies

	P-FNA	USG-FNA	*p*-value
Nodule number (n)	78	190	
Age (years)*	47 ± 15	47 ± 12	0.86
Men (%)†	14 (18%)	42 (22%)	0.45
Euthyroid (%)†	69 (89%)	158 (83%)	0.27
Hypothyroid (%)†	5 (6%)	20 (11%)	0.29
Hyperthyroid (%)‡	4 (5%)	12 (6%)	1.00
Right lobe (%)†	39 (50%)	84 (44%)	0.48
Isthmus (%)†	12 (15%)	25 (13%)	0.48
Log (nodule diameter)*§	1.26 ± 0.18	1.27 ± 0.19	0.58
Malignant (%)‡	2 (3%)	6 (3%)	1.00
Benign (%)†	38 (49%)	118 (62%)	0.04
Indeterminate (%)‡	5 (6%)	11 (6%)	0.78
Inadequate (%)†	33 (42%)	55 (29%)	0.03

Each case is a nodule, not a subject. P-FNA: palpation-guided thyroid fine-needle aspiration biopsy, USG-FNA: ultrasound-guided thyroid fine-needle aspiration biopsy *mean and standard deviation are given and Student's *t *test is used to compare groups †frequency and percentage are given and χ^2 ^test is used to compare groups ‡frequency and percentage are given and Fisher's exact test is used to compare groups. §logarithm of maximal nodule diameter expressed as log (mm).

**Table 3:** Cross-tabulation of cytology and surgical histopathology results in palpation-guided thyroid fine-needle aspiration biopsies

	Disease positive	Disease negative
Test positive	1	3
Test negative	1	6

Disease positive: malignant surgical histopathology, Disease negative: benign surgical histopathology, Test positive: malignant or indeterminate cytology result, test negative: benign or unsatisfactory cytology result.

**Table 4:** Cross-tabulation of cytology and surgical histopathology results in ultrasound-guided thyroid fine-needle aspiration biopsies

	Disease positive	Disease negative
Test positive	8	3
Test negative	2	5

Disease positive: malignant surgical histopathology, Disease negative: benign surgical histopathology, Test positive: malignant or indeterminate cytology result, test negative: benign or unsatisfactory cytology result.

**Figure 1:**
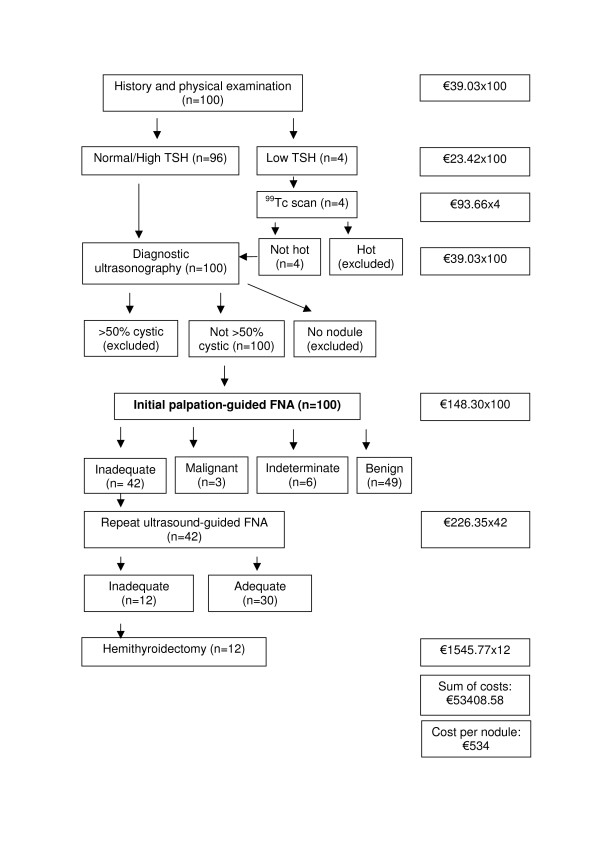
**Cost calculation of palpation-guided thyroid fine-needle aspiration biopsy strategy**. Cost of diagnostics steps were summed until a final diagnosis was established for one hundred thyroid nodules for which initial palpation-guided thyroid fine-needle aspiration biopsy was performed by simulating American Thyroid Association thyroid nodule management guideline. Strategy cost was calculated by dividing the total cost to 100. FNA: fine-needle aspiration biopsy.

**Figure 2:**
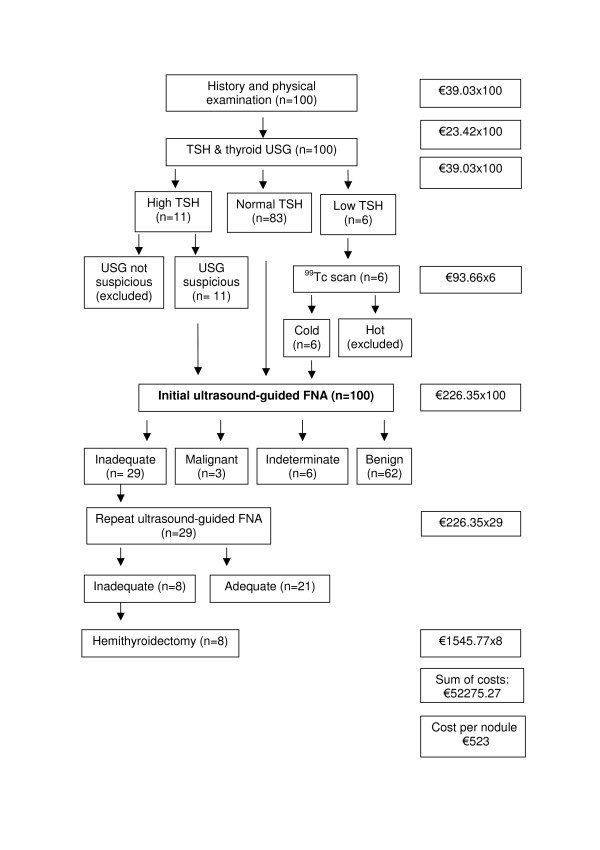
**Cost calculation of ultrasound-guided thyroid fine-needle aspiration biopsy strategy**. Cost of diagnostic steps were summed until a final diagnosis was established for one hundred thyroid nodules for which initial ultrasound-guided thyroid fine-needle aspiration biopsy was performed by simulating American Association of Clinical Endocrinologists thyroid nodule management guideline. Strategy cost was calculated by dividing the total cost to 100. FNA: fine-needle aspiration biopsy, USG: ultrasonography.

Figure 1 shows that P-FNA strategy costs €534 per nodule, if one follows ATA thyroid nodule management guidelines. As estimated in the previous paragraph, P-FNA correctly classifies 64% of palpable, non-cystic thyroid nodules as benign versus malignant. Figure 2 shows that USG-FNA strategy costs €523 per nodule, if one follows AACE guidelines. As shown in the previous paragraph, USG-FNA correctly classifies 72% of palpable, non-cystic thyroid nodules as benign versus malignant. ICER is -€138. The negative ICER means that utilization of USG-FNA instead of P-FNA results in a saving of 138 Euros for one additional correct diagnosis of thyroid malignancy.

## Discussion

Although initial cost of USG-FNA is higher, this study shows that universal USG-FNA for all thyroid nodules is cost-effective compared to P-FNA strategy. Existing thyroid nodule management guidelines were simulated, an approach not followed in previous studies. Other researchers showed that thyroid FNA is cost-effective compared to thyroid scintigraphy or to diagnostic ultrasonography in detecting thyroid cancer [17,18]. This is the only study that shows USG-FNA is cost-effective compared to P-FNA. Previous studies showed that USG-FNA strategy is expensive than P-FNA strategy [5,19], meaning that although more accurate, USG-FNA is not cost-effective compared to P-FNA. Cesur et al. reported that USG-FNA is €13 expensive than P-FNA [5]. Khalid and coworkers found that USG-FNA costs €184 more for one additional correct diagnosis [19]. Existing data for cytology results were compiled from the literature in that study. In Khalid and coworkers' study, the inadequacy rate of P-FNA was 20%, the inadequacy rate of USG-FNA was 5%, the accuracy rate of P-FNA was 0.92 and the accuracy rate of USG-FNA was 0.98 [19]. These were somewhat lower inadequate and higher accuracy rates than found in this study and in studies given in table 5. When the inadequate biopsy rate is low, like the studies of Danese et al. [3], Izquierdo et al. [6] and Khalid et al. [19], USG-FNA is not cost-effective over P-FNA and ICER, the extra cost to achieve one more accurate diagnosis is over €250 (table 5). In other studies with higher inadequate rates [4,5], ICER ranged between €58 and €90 (table 5). If the use of harmonic scalpel becomes a standard in thyroid surgery [20], the cost of thyroid surgery will increase. Khalid and coworkers reported that when the cost of surgery increases, USG-FNA becomes more cost-effective [19].

**Table 5:** Cost-effectiveness comparison between palpation- and ultrasound-guided thyroid fine-needle aspiration biopsies with costs from this study and outcomes from previous studies

Author Year	n	P-FNA Inadequate	P-FNA Strategy Cost*	P-FNA Accuracy	USG-FNA Inadequate	USG-FNA Strategy Cost*	USG-FNA Accuracy	ICER	Reference
Danese 1998	9683	9%	€270	0.73	4%	€337	0.76	+€2233	[3]
Hatada 1998	166	30%	€395	0.48	17%	€413	0.68	+€90	[4]
Cesur 2006	285	32%	€430	0.77	21%	€437	0.89	+€58	[5]
Izquierdo 2006	376	11%	€290	0.61	7%	€344	0.80	+€284	[6]
Can 2009	268	42%	€534	0.64	29%	€523	0.72	-€138	This study

n: total number of nodules, P-FNA: palpation-guided thyroid fine-needle aspiration biopsy, Inadequate: inadequate rate, USG-FNA: ultrasound-guided thyroid fine-needle aspiration biopsy, ICER: incremental cost-effectiveness ratio. ICER is calculated by dividing the difference between strategy cost of USG-FNA and strategy cost of P-FNA to the difference between accuracy of USG-FNA and P-FNA. A positive ICER value indicates more, a negative ICER value indicates less expense for USG-FNA strategy over P-FNA strategy *Strategy cost is derived from figure 1 or 2, table 1 and cytology outcomes from references.

### Manipulation of the data with alternatives

If the diagnostic step of hemithyroidectomy (n = 12 for P-FNA and n = 8 for USG-FNA) is excluded from Figures 1 &2, the average cost of P-FNA will be €349 and USG-FNA €399 in this study. As the analyses reported here are subject to substantial changes depending on the variation of costs and FNA outcomes, alternative data sources are used. The first approach is to replace private health care costs that are used in this study (table 1) with state hospitals' costs in Turkey. Turkish Ministry of Health owns and operates state hospitals and licenses and inspects private hospitals. Turkish Ministry of Labor and Social Security manages Turkish Social Security Institution which covers 80% of the population and is the main payer for health care [21]. All state hospitals provide services to patients insured by the Turkish Social Security Institution from the prices that the Institution determines. Turkish Social Security Institution's prices are obtained from the internet [21] and Ankara University, Faculty of Medicine (Murat Faik Erdogan, MD, murat.erdogan@temd.org.tr, e-mail communication, June 10, 2008). These prices were valid for all state hospitals in Turkey in 2008. Following are Turkish State Hospitals' fees: history and physical exam €8, TSH measurement €2.32, thyroid ultrasonography €7.49, Tc^99m ^thyroid scintigraphy €9.65, P-FNA €36.81, USG-FNA €52.14, subtotal hemithyroidectomy €393.76. From a perspective of Turkish Social Security Institution prices and FNA outcomes of this study, P-FNA strategy costs €124 and USG-FNA strategy costs €117 for one thyroid nodule. ICER is -€88, again indicating savings with USG-FNA. The next example is to input other authors' costs to our thyroid FNA outcomes. From a mid-Atlantic academic medical center in the USA, the cost of initial P-FNA was reported as €162, USG-FNA as €263 and hemithyroidectomy as €1733 [19]. These were hospital costs and were not actual billed charges. When these costs are inputted into the last five boxes of Figure 1 and 2, the cost of P-FNA strategy is estimated as €480 and the cost of USG-FNA strategy as €478. ICER is -€25, indicating savings with USG-FNA strategy. The other approach is to use costs in table 1 with inadequate and accuracy rates from the literature. A cost-effectiveness comparison is made by using data from previous studies that report both the accuracy rates and head-to-head comparison of P-FNA with USG-FNA and is illustrated in table 5.

### Limitation of the study

A cystic nodule was defined if the cystic component is more than 90% of nodule area in this study. ATA recommends USG-FNA if a nodule is more than 50% cystic [1]. Posterior location of the nodule, an indication for USG-FNA was not recorded and could not be used in the construction of ATA thyroid nodule management guidelines. Another point to remember is that actual costs should be approximately 30% lower in this study, because charges for diagnostic steps, like physical examination or TSH are for per patient, not for per nodule. There were four nodules with medullary thyroid cancer from three patients in the USG-FNA group. A genetic RET screening was requested. It was refused by one subject, the other subject was lost to follow-up and RET analysis is pending in the third subject. The medullary thyroid cancer patients were not from the same family. A single familial cluster of medullary thyroid cancer was not present. The assignment to P-FNA or USG-FNA groups was not affected by the presence of medullary thyroid cancer and there was no bias in the study from that aspect. Our inadequacy rates are high (42% for P-FNA and 29% for USG-FNA). Mehrotra and coworkers reported an inadequacy rate of 47% for P-FNA and 16% for USG-FNA [9]. Our inadequacy rates are not greatly different from Mehrotra's rates. Cytology outcomes of this study reflect everyday clinical practice. This may be an advantage in a cost-effectiveness comparison, because controlled studies may have different outcomes than real-life situations.

## Conclusion

From a perspective of ATA and AACE thyroid nodule management guidelines and with costs from Turkish health care system, this study shows that USG-FNA is cost-effective. If universal USG-FNA is used instead of P-FNA, 138 Euros are saved for one additional accurate diagnosis of benign versus malignant thyroid nodular disease.

## Abbreviations

AACE: American Association of Clinical Endocrinologists; ASC: Ahmet Selçuk Can (the author of this study); ATA: American Thyroid Association; FNA: fine-needle aspiration biopsy; ICER: incremental cost-effectiveness ratio; P-FNA: palpation-guided thyroid fine-needle aspiration biopsy; USG-FNA: ultrasound-guided thyroid fine-needle aspiration biopsy.

## Competing interests

The author declares that he has no competing interests.

## Authors' contributions

ASC is the sole author of this manuscript.

## Pre-publication history

The pre-publication history for this paper can be accessed here:


